# Unusual terahertz waveforms from a resonant medium controlled by diffractive optical elements

**DOI:** 10.1038/s41598-019-43852-w

**Published:** 2019-05-15

**Authors:** A. V. Pakhomov, R. M. Arkhipov, M. V. Arkhipov, A. Demircan, U. Morgner, N. N. Rosanov, I. Babushkin

**Affiliations:** 1 St. Petersburg State University, 7/9 Universitetskaya nab., St. Petersburg, 199034 Russia; 2 ITMO University, Kronverkskiy Prospekt 49, St. Petersburg, 197101 Russia; 3 Ioffe Institute, Politekhnicheskaya str. 26, St. Petersburg, 194021 Russia; 4 Institute of Quantum Optics, Welfengarten 1, 30167 Hannover, Germany; 5 Cluster of Excellence PhoenixD (Photonics, Optics, and Engineering – Innovation Across Disciplines), Hannover, Germany; 6 Vavilov State Optical Institute, Kadetskaya Liniya v.o. 5/2, St. Petersburg, 199053 Russia; 7 Max Born Institute, Max-Born-Strasse 2a, Berlin, 10117 Germany

**Keywords:** Ultrafast photonics, Terahertz optics, Nonlinear optics

## Abstract

Up to now, full tunability of waveforms was possible only in electronics, up to radio-frequencies. Here we propose a new concept of producing few-cycle terahertz (THz) pulses with widely tunable waveforms. It is based on control of the phase delay between different parts of the THz wavefront using linear diffractive optical elements. Suitable subcycle THz wavefronts can be generated via coherent excitation of nonlinear low-frequency oscillators by few-cycle optical pulses. Using this approach it is possible to shape the electric field rather than the slow pulse envelope, obtaining, for instance, rectangular or triangular waveforms in the THz range. The method is upscalable to the optical range if the attosecond pump pulses are used.

## Introduction

Generation of few-cycle pulses in terahertz (THz) (0.1–10 THz) range is a subject of intensive research^1–5^. Large molecules have vibrational and rotational transitions in this region, which makes such pulses important in high resolution spectroscopy, biological sensing, medicine, wireless communication systems etc^5–7^.

Most of the methods of THz pulse generation proposed up to now are based on a nonlinear conversion of optical pulses in nonlinear media^1,2^ using optical rectification in different media such as lithium niobate^8,9^, semiconductors^10,11^ and organic crystals^12,13^, or the ionization-based nonlinearity (so called Brunel mechanism^14^) which leads to creation of plasma currents in gases^15–18^ or semiconductors^1,2^ (in the latter case the electric current is created via transition of electrons from valence to conducting bands). The latter idea was further developed by using photoconductive nanoantennas^5,19^.

Currently, there is a big interest in half-cycle pulses at various wavelengths^20–24^ since they are important in ultrafast spectroscopy^23,25^, as well as in attosecond science^26,27^. For instance, they are in certain sense more efficient in excitation of quantum oscillators^23,28^. Typically, half-cycle pulses contain a single strong burst of electromagnetic field and a long low-amplitude tail of the opposite polarity. This specific feature of subcycle pulses makes them an attractive tool to control electron wavepacket dynamics^23,28^. Such pulses are sometimes called quasi-unipolar^29^, taking in mind truly unipolar pulses, which, according to some theoretical predictions, is possible to obtain as a result of interaction of light with resonant media^24,29–40^, or as the result of charged particle acceleration^41,42^.

Subcycle pulses can be especially easily obtained in the THz range^1,2^, but even in the optical range such pulses are becoming now available^20–24^. Different methods of unipolar and quasi-unipolar pulse generation via accelerated electric charges^41,42^, half-cycle solitons in nonlinear medium^30–35^, reflection of single-cycle pulse from nonlinear film^24^ as well as in media with different types of nonlinearity^29,36–40^ were recently discussed.

Another important problem in few-cycle pulse generation is pulse shaping. Generation of arbitrary optical waveforms is an important issue in many applications such as coherent control of quantum and nonlinear processes, and lightwave communications^43^. For THz pulse shaping and phase control different schemes have been proposed, see refs^44–49^ and references therein. These methods enable control of THz pulses by introducing dispersion elements^44^, prism wave plates^45^, parallel-plate waveguides^46,47^ or by the pump pulse shaping^48,49^.

An alternative recent approach is based on a coherent excitation and control of the free induction decay^50^ in a medium with ultra-short optical pulses^29,38–40^. In this approach, nonlinear dipoles with a resonance at low frequencies (THz or MIR ranges) are to be excited by pairs of ultrashort pulses in optical range, one of them starting the slow THz-scale oscillations of the dipoles and another stopping it^38,39^. Between the optical pulses, a slow oscillator radiates via free induction decay^50^, which allows to generate subcycle quasi-unipolar pulses. Radiation from many of such oscillators located at different spatial positions, being summed with a certain phase delay, resulted in tunable waveforms^40^ appearing at the detector. This method requires the control of spatio-temporal phase delays of partial waves from different oscillators. Methods to control these phases proposed up to now were rather exotic and difficult to realize; for instance, it was suggested to make a thin string of an active medium, illuminated by a spot of light which moves along the string with a superluminal velocity^51,52^. Such approach, being difficult to realize in practice, provides only limited degree of control over the waveshape of the generated pulse.

Here we introduce a much more affordable approach based on linear diffractive optical elements (DOE), which allows to control the phases of the partial waves coming to the detector from different spatial positions and thus to shape the resulting THz waveform. In contrast to many other approaches, our method allows to control the electric field directly rather than its slow envelope, allowing to obtain exotic waveshapes such as rectangular or triangular ones.

## The Setting

**Figure 1:**
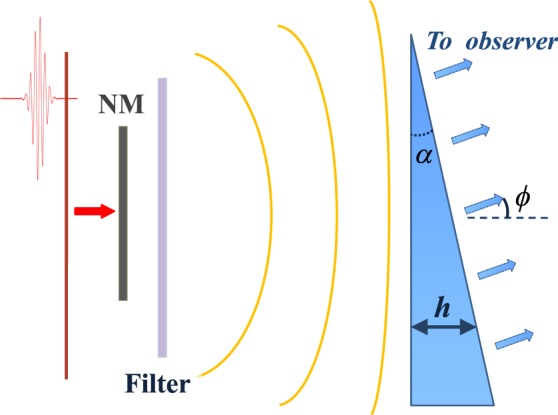
The scheme of the setup: a thin layer composed of a nonlinear medium (NM) with a resonance in THz range is excited by two ultrashort pulses (for clarity only one of the pulses is shown as a red curve). The radiation is then passed through a low-pass filter and then through a diffractive optical element (e.g., triangle-shaped wedge, which is shown here) and is sent to a detector.

**Figure 2:**
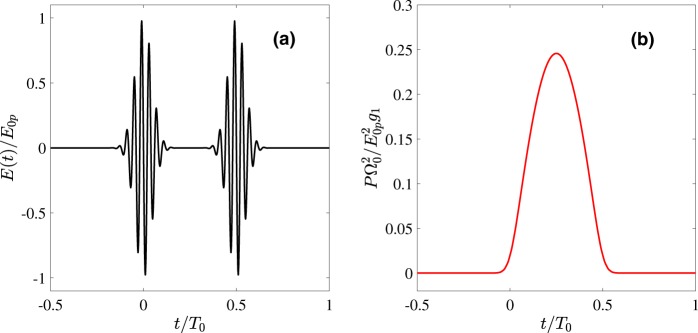
(**a**) Two driving Gaussian pulses (see Eq. (9)) with the optical frequency *ω*_0_ = 25Ω_0_ and pulse duration *τ*_0_Ω_0_ = 0.5. The driver pulses are separated by the time interval *τ*_*p*_ = *T*_0_/2; Here *T*_0_ = 2*π*/Ω_0_ = 10^−13^ *s*. (**b**) A single oscillation of the polarization of the medium — initiated by the first driver pulse and stopped by the second one.

The idea of the proposed method is to generate subcycle THz waveforms in a thin nonlinear layer (see Fig. 1) and then phase-shape the partial waves on the way to the detector. We assume that the nonlinear layer possesses a low-frequency resonance in the THz range. This resonance is excited by a pair of strong few-cycle pulses at the optical frequency. The first pulse, as it is shown in Fig. 2, starts the low-frequency oscillators whereas the second stops it. In between the pulses, the oscillators radiate via free polarization decay mechanism^50^. After the layer, we place a diffractive optical element (DOE), which influences the phases of the THz waves coming from different positions of the layer, and thus shapes the THz waveform at the detector.

The light propagation in the system is governed by the wave equation:

1 $${\rm{\Delta }}E(\overrightarrow{r},t)-\frac{1}{{c}^{2}}\frac{{\partial }^{2}E(\overrightarrow{r},t)}{\partial {t}^{2}}=\frac{4\pi }{{c}^{2}}\frac{{\partial }^{2}P(\overrightarrow{r},t)}{\partial {t}^{2}},$$

where $P(\overrightarrow{r},t)$ is the polarization of the medium, $E(\overrightarrow{r},t)$ is the electric field. We consider for simplicity the configurations where all fields are linearly polarized in the same direction, in which case a scalar version of the wave equation can be used.

We suppose furthermore the propagation everywhere except the thin nonlinear layer NM in Fig. 1 to be linear. The layer NM is to possess a low-frequency resonance Ω_0_ = 2*π*/*T*_0_ in THz or MIR range. This can be, for instance, a Raman-active medium^53,54^, a set of nonlinearly coupled plasmon resonances in metallic nanoparticles^55^, nanoantennas^56,57^, quantum dots^58^, and others. Of a special interest to date are novel types of materials such as graphene or topological insulators^59–61^ which also have strong broadband nonlinear response in the THz range^60^. The polarization *P* in the nonlinear layer can be described by:

2 $$\ddot{P}+\gamma \dot{P}+{{\rm{\Omega }}}_{0}^{2}P=g[E(t)]E(t),$$

where Ω_0_ and *γ* are the resonant frequency and decay rate, respectively, and the function *g*[*E*(*t*)] describes the nonlinear coupling of the oscillators to the pump field. We assume that the frequency of the pump field is in the optical range, that is, far away from Ω_0_. Because of the non-resonant excitation, the dynamics of populations in the layer can be neglected. We also assume that the molecules/particles are asymmetric and pre-aligned so that their coupling to the field can be described in lowest order by a quadratic nonlinearity^38^: *g*[*E*] = *g*_1_*E*. Such response is common in nonlinear crystals with a quadratic nonlinearity. However, here we are interested in the low-frequency response rather than the instantaneous one. Eq. (2), coupled to Eq. (1) describes the light propagation and generation of new frequencies in the nonlinear layer. In particular, the slow oscillators, being excited by ultrashort optical pulses, can radiate after the pump is gone via free induction decay^50^.

In the next subsections we proceed by consideration of (i) a response of a single oscillator in the layer to the pair of the pulses in Fig. 2 and estimating the nonlinear propagation effects in the layer, (ii) propagation of the partial waves from every of the oscillator to the DOE, (iii) modification of the THz radiation inside the DOE, and (iv) its propagation to the detector as shown in Fig. 1. Furthermore, in the subsequent sections we consider some specific examples of the media and DOEs, determining the resulting THz field amplitude.

### Response of a single oscillator

It is natural to start our description from a single nonlinear oscillator. Suppose that an oscillator with the response defiend by Eq. (2) is excited by an ultrashort pulse at the optical frequency, so that the duration of the driving pulse *τ*_0_ is much smaller than the period of the resonant oscillations in the medium *T*_0_ = 2*π*/Ω_0_. Since we are dealing here with few-cycle pulses, we can neglect the damping of the oscillators in Eq. (2), that is, we assume *γ* = 0. As it was shown in^40^, under such excitation Eq. (2) can be integrated to give the low-frequency response of the oscillator as:

3 $$\begin{array}{c}P(t)=\frac{\sin ({{\rm{\Omega }}}_{0}t)}{{{\rm{\Omega }}}_{0}}{\int }_{-\infty }^{t}g[E(t^{\prime} )]E(t^{\prime} )\cos ({{\rm{\Omega }}}_{0}t^{\prime} )dt^{\prime} \\ \,\,\,-\frac{\cos ({{\rm{\Omega }}}_{0}t)}{{{\rm{\Omega }}}_{0}}{\int }_{-\infty }^{t}g[E(t^{\prime} )]E(t^{\prime} )\sin ({{\rm{\Omega }}}_{0}t^{\prime} )dt^{\prime} .\end{array}$$

Here the integration is performed over the whole pump pulse duration and beyond. The second term becomes negligible in Eq. (3) if a symmetric temporal shape of the excitation pulse^40^ is assumed. Thus, our simplified equation Eq. (3) shows that after the passage of the pump pulse the polarization of the medium continues to oscillate: *P*(*t*) = Π_0_sin(Ω_0_*t*) with ${\Pi }_{0}=\frac{1}{{{\rm{\Omega }}}_{0}}{\int }_{-\infty }^{+\infty }g[E(t^{\prime} )]E(t^{\prime} )\cos ({{\rm{\Omega }}}_{0}t^{\prime} )dt^{\prime}$.

Oscillations of the polarization in turn lead to emission due to free induction decay^50^. In order to generate a quasi-unipolar pulse, the oscillations of polarization initiated by the first driver pulse have to be stopped by another (identical) pulse positioned at the distance *τ*_*p*_ = *T*_0_/2 to the first one (see Fig. 2a). That is, the oscillator makes a half of its own proper oscillation before it is stopped by the “counter-pulse”. Figure 2b illustrates the resulting temporal shape of the polarization scaled to ${g}_{1}{E}_{0p}^{2}{/{\rm{\Omega }}}_{0}^{2}$.

The field created by a single oscillator and observed at the distance *D* is then given by the well-known solution of the wave equation Eq. (1) in the dipole approximation^62^:

4 $$E(t)=\frac{\ddot{P}(t)}{{c}^{2}D}.$$

**Figure 3:**
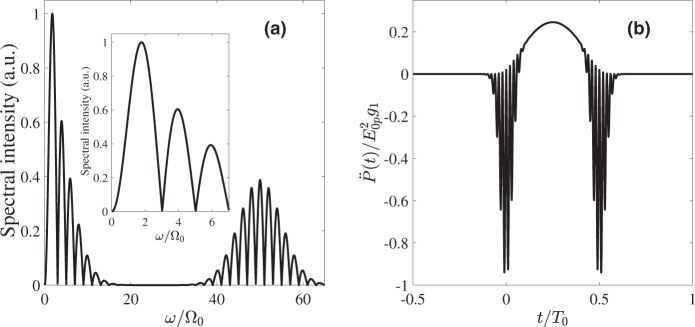
(**a**) The spectrum of a typical response, according to Eq. (4), of an oscillator governed by Eq. (2) (see Fig. 2b); The parameters are the same as in Fig. 2. The inset shows the magnified low-frequency part of the spectrum. (**b**) The temporal waveshape corresponding to (a). The central half-sine wave of duration *T*_0_/2 is surrounded by high-frequency oscillations at the pulse edges arising due to the action of the pump pulses which are to be removed with the low-pass filter.

Figure 3 shows an example of the field produced by an oscillator Eq. (2) under the excitation by two Gaussian pump pulses. Its temporal profile is shown in Fig. 3b and consists of a central low frequeny half-sine wave as well as high frequency oscillations during the action of the pump pulses. Thus, the spectrum of the field shown in Fig. 3a consists of two distant parts, the low-frequency one corresponding to the half-oscillation at Ω_0_ whereas the high-frequency one corresponds to the co-oscillations with the driver pulse. This spectral separation allows us to get rid of high-frequency components by an appropriate low-pass filter, thus keeping only low-frequency part of the spectrum in Fig. 3a. The spectrum does not contain a dc-component, i.e. the resulting pulse is not unipolar. However, proper shaping of the filter as described below allows to transform the pulse in Fig. 3b into the quasi-unipolar one, with long low-amplitude tails surrounding the central intense burst. The appropriate filter should cut off the frequencies with *ω* > *ω*_*cut*_ ≫ Ω_0_. That is, its spectral transmission function *Tr*(*ω*) of the filter should have the following form:

5 $$Tr(\omega )=F[\omega ]\cdot {\rm{\Theta }}[{\omega }_{cut}-\omega ],$$

where Θ is the Heaviside step-function, *ω*_*cut*_ is the cut-off frequency and a smooth function *F*[*ω*] has maximum around Ω_0_. The function *F*[*ω*] is selected so that the waveshape possibly nearest to a single half-waveshape such as shown in Fig. 2b appears and its particular form depends on the nonlinear coupling function of the specific medium. More detailed description of *F*(*x*) is given in the next subsection.

Assuming then that the high-frequency oscillations are filtered out, the emitted radiation *E*(*t*) has the form of a quasi-unipolar pulse^38–40^ and without regard to the lateral tails, can be approximated as the sum of waves of the form Eq. (3) giving a half of a sinusoidal wave:

6 $$E(t)=\tilde{E}\sum _{k=0}^{1}\,{\sin {\rm{\Omega }}}_{0}{t}_{k}\cdot {\rm{\Theta }}[{t}_{k}],$$

where *t*_*k*_ = *t* − *kτ*_*p*_, and the effective scaling constant $\tilde{E}$ is given as: $\tilde{E}=\frac{{{\rm{\Omega }}}_{0}^{2}{{\rm{\Pi }}}_{0}}{{c}^{2}D}$.

### Molecular model

In Eq. (2) we assumed a model of a general low-frequency oscillator with a quadratic coupling to the IR field. Here we consider a more specific model representing a molecule with the dynamics of the nucleus and the electrons taken into account explicitly^53,54^ (we will call it a Raman-active medium (RAM) model). The model is classical and consists of two nonlinearly-coupled oscillators with strongly different resonant frequencies, which we call the high-frequency oscillator (HFO) and low-frequency oscillator (LFO), respectively. The role of HFO and LFO are played by the electrons and nuclei in the molecule^54^. Let *y*, *x* denote the normal coordinates of LFO and HFO oscillations. The potential energy of the nonlinearly-bonded oscillators in RAM is given as^53,54^:

$$U(x,y)=\frac{1}{2}\alpha {x}^{2}+\frac{1}{2}\beta {y}^{2}+\frac{1}{2}\gamma {x}^{2}y,$$

where *α* and *β* stand for the elastic constants of the both oscillators, factor *γ* gives the strength of the nonlinear bonding between HFO and LFO. The equations for the dynamics of RAM under a pulsed excitation take the following form^53,54^:

7 $$\ddot{x}+{{\rm{\Gamma }}}_{e}\dot{x}+{\omega }_{e}^{2}x=\frac{q}{m}{E}_{p}(t)-\frac{\gamma }{m}xy,$$

8 $$\ddot{y}+{{\rm{\Gamma }}}_{n}\dot{y}+{{\rm{\Omega }}}_{0}^{2}y=-\frac{\gamma }{2M}{x}^{2}.$$

Here, *M* and *m* are effective masses of the LFO and HFO, *E*_*p*_(*t*) is the pump electric field, Γ_*e*_ and Γ_*n*_ are the damping rates of HFO and LFO, respectively, ${{\rm{\Omega }}}_{0}=2\pi /{T}_{0}=\sqrt{\frac{\beta }{M}}$ is the resonance frequency of LFO (nuclei), ${\omega }_{e}=2\pi /{T}_{e}=\sqrt{\frac{\alpha }{m}}$ is the natural frequency of the HFO (electrons). One can see that this model is to some extend equivalent to Eq. (2). Namely, the displacement of the nuclei in Eq. (8) is driven by the term proportional to *x*^2^ and hence, due to large ratio of the resonant frequencies of LFO and HFO, the effective equation for LFO can be reduced to Eq. (2).

We suppose that the medium is excited by two Gaussian pump pulses:

9 $${E}_{p}(t)={E}_{0p}\,\exp [-{t}^{2}/{\tau }_{0}^{2}]\,\sin \,{\omega }_{0}t+{E}_{0p}\,\exp [-{(t-{\tau }_{p})}^{2}/{\tau }_{0}^{2}]\,\sin \,[{\omega }_{0}(t-{\tau }_{p})],$$

separated by the time interval *τ*_*p*_ = *T*_0_/2. The polarization of the medium for such ultrashort pump pulses can not be adequately described anymore in terms of molecular polarizability and we apply the general definition:

10 $$P=Nd=qN(x+y),$$

where *d* is the dipole moment of a single RAM molecule (composed of two coupled oscillators), *N* is the concentration of molecules and the charges of both oscillators *q* are equal in magnitude due to charge neutrality of the medium.

**Figure 4:**
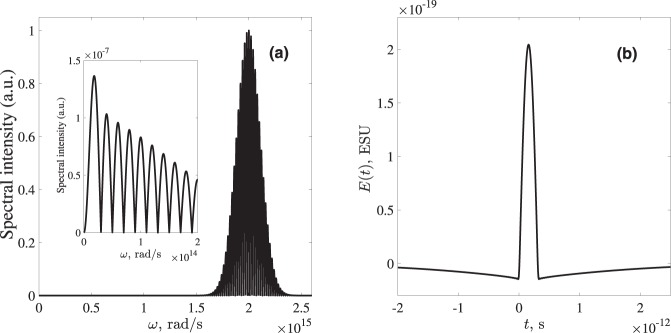
(**a**) The spectrum and (**b**) the temporal waveshape of the molecular response according to the model Eqs (7–8), obtained for the parameters described in the text after passing through the filter [Eqs. (5) and (11)]. The inset in (**a**) shows the low-frequency part of the spectrum magnified.

Figure 4a shows the spectrum of the pulse obtained using Eq. (4) with the following parameters: Ω_0_ = 10^13^ rad/s, *ω*_*e*_ = 10^15^ rad/s, *m* = 9.1 · 10^−28^ g (electron mass), *M* = 1.6 · 10^−24^ g (proton mass), electric charge *q* = 4.8 · 10^−10^ ESU, *γ* = 10^8^ erg/cm^3^, Γ_*e*_ = 10^14^ s^−1^, Γ_*n*_ = 0 s^−1^, *E*_0*p*_ = 10^5^ ESU, *τ*_0_ = 12.5 fs, *ω*_0_ = 2 · 10^15^ rad/s.

**Figure 5:**
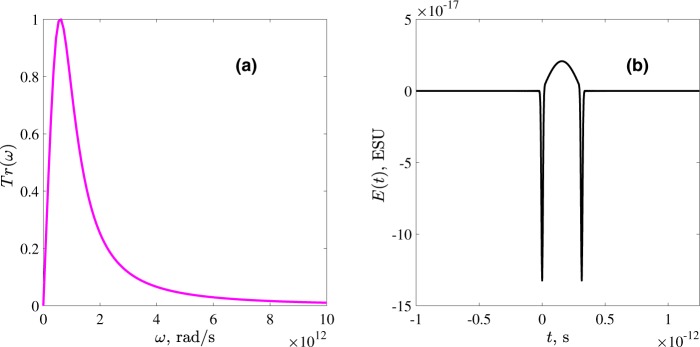
(**a**) The spectral transmission function *Tr*(*ω*) of the filter Eqs (5) and (11); (**b**) The temporal waveshape of the pulse from a single RAM molecule Eq. (4) after passing through the filter with *F*(*ω*) = 1 in Eq. (11).

The low-frequency part of the spectrum obtained with this more detailed model has a significantly lower amplitude but also somewhat different spectrum than the one based on the more simple Eq. (2) (as given in Fig. 3). To find the function *F* in the filter *Tr*(*ω*) making the response at the detector from single oscillator as “unipolar” as possible, we first notice that the envelope of the half-sine wave has the form of (*ω*/Ω_0_)^−2^ for $\omega \gtrsim {{\rm{\Omega }}}_{0}$. At the same time, the low-frequency part of the spectrum in Fig. 4a is decreasing much slower. Based on these arguments, the function *F* determining the properties of low-pas filter Eq. (5) at low frequencies has the following form:

11 $$F(\omega )=\frac{{\mu }_{1}[\frac{\omega }{{{\rm{\Omega }}}_{0}}]}{1+{({\mu }_{2}\frac{\omega }{{{\rm{\Omega }}}_{0}})}^{3}},$$

where the factors *μ*_1_ and *μ*_2_ control the maximum of the filter transmittance and its bandwidth respectively. In Eqs (5) and (11) we take *ω*_*cut*_ = 10^14^ rad/s and *μ*_1_ = 25.26, *μ*_2_ = 13.33. The resulting transmittance function is shown in Fig. 5a.

The waveshape of the pulse from a single RAM molecule according to Eq. (4) after passing through the filter is shown in Fig. 4b. The observer is assumed to be located at the distance *D* = 10 cm ≫ *R*. It is seen that the pulse after the filter has the shape of a half-sine wave of the duration *T*_0_/2 = 3.14 · 10^−13^ s^−1^ surrounded by weak long tails, which fully compensate the electric area of the central part, giving zero value of the dc-component. However, it can be easily shown (see Appendix III) that taking into account these long tails results just in a minor modification of the amplitude of the pulse after an arbitrary DOE without changing the main part of its waveform.

As said, the form of the filter Eq. (5) is taken to smooth out the oscillations at the edges of the pulse. To illustrate this in more details we plot in Fig. 5b the pulse shape from a single RAM molecule Eq. (4) after passing through a simple low-pass step filter *Tr*(*ω*) = Θ[*ω*_*cut*_ − *ω*], with the same value of *ω*_*cut*_. One can see that, although the high-frequency oscillations at the edges of the central half-sine wave are indeed filtered out, the emitted pulse still possesses sharp peaks at the edges. These peaks make the phase-delay control procedure we consider later more involved. Therefore we apply a filter with relatively narrow transmission bandwidth at low frequencies which turns these sharp peaks into the low-amplitude long tails in Fig. 4b.

### Estimation of the generated THz field and of the nonlinear side-effects

Even before we start considering the details of propagation it is reasonable to estimate the amplitude of the THz wave at the detector produced by the whole layer as well as the nonlinear propagation effects. For this, we assume the shape of the nonlinear layer to be a disk of the radius *R* = 1 cm, thickness *h*_*L*_ = 0.1 mm and concentration of oscillators *N* ≈ 5 · 10^22^ cm^−3^. To achieve a reasonable THz field strength, the pump field in the RAM model described in the previous section should be taken to be 10^5^ ESU which corresponds to around 1 TW/cm^2^ intensity. The pulse with such large radius and intensity should have the energy of about 50 mJ. Nevertheless the big radius (much larger than the wavelength of THz radiation) is absolutely necessary for the following shape control. The total number of oscillators is *N*_0_ = *NπR*^2^*h*_*L*_ ≈ 1.5 · 10^21^. The amplitude at the detector can be estimated as a sum over all the oscillators as ${E}_{\Sigma } \sim {N}_{0}\tilde{E}$ which gives for these parameters around ≈300 ESU ≈100 kV/cm, which is comparable to the amplitudes generated by other methods.

We remark that the width of our layer is much less than THz wavelength so that we can neglect the THz diffraction in the layer. Besides, the THz wave is still too weak to demonstrate nontrivial nonlinear propagation effects on this length scale. The nonlinear propagation effects of the pump pulse can be also easily estimated and are still negligible for these parameters. Namely, for such a large pulse diameter we have the characteristic nonlinear distance *n*_2_*ω*_0_*I*/(*cS*) ≈ 100 m (here *n*_2_ is the nonlinear coefficient which we take as *n*_2_ = 3 × 10^−16^ cm^2^/W for SiO_2_ for simplicity, *S* is the area of the beam and *I* is the intensity which is in our case around 1 TW/cm^2^). The absorption of the pump wave can be estimated as the following: the part of the excited atoms is estimated as *μ* ≈ (Ω_0_/*ω*_0_)^2^ ~ 10^−4^. Therefore the energy *μN*_0_ℏΩ_0_ absorbed from the pump should be around ≈0.5% of the pump energy and thus the absorption of the pump can be also neglected in the first approximation.

### Propagation through DOE

The pulse in Fig. 3 obtained for Eq. (2) or alternatively from Eqs (7–10) actually gives the emission from a single oscillator. In the previous section we made a raw estimation of the generation efficiency by a simple summation of impacts of all oscillators without taking into account diffraction effects.

In the following we sum the impacts of all oscillators in a more rigorous way including all the elements in Fig. 1. We consider the further propagation to be linear and therefore described by a standard diffraction theory^62^. As a first step, we calculate the field just in front of the DOE (see Fig. 1). We assume that the distance between the DOE and the layer is significantly larger than the transverse size of the layer. We denote the entrance plane of DOE as *z* = *z*_0_ (while *z* = 0 corresponds to the surface of the layer) and ${\overrightarrow{r}}_{\perp }$ is the vector in this plane. The wave equation Eq. (1) can be integrated to give the field $E({\overrightarrow{r}}_{\perp },z={z}_{0},t)$ as^62^:

12 $$\overrightarrow{E}({\overrightarrow{r}}_{\perp },z={z}_{0},t)=\frac{1}{{c}^{2}\tilde{D}}{\iiint }_{NM}\frac{{\partial }^{2}\overrightarrow{P}}{\partial {t}^{2}}(\overrightarrow{r}^{\prime} ,t-\frac{\tilde{D}}{c}+\frac{\overrightarrow{r}^{\prime} \overrightarrow{u}^{\prime} }{c}){d}^{3}\overrightarrow{r}^{\prime} ,$$

where $\tilde{D}$ is the distance between the center of the medium layer and point ${\overrightarrow{r}}_{\perp }$: $\tilde{D}=\sqrt{{z}_{0}^{2}+{\overrightarrow{r}}_{\perp }^{2}}$, the integration is performed over the whole volume of the nonlinear medium (NM) and the unit vector $\overrightarrow{u}^{\prime}$ is pointing from the center of the layer to the point ${\overrightarrow{r}}_{\perp }$. Let us assume that we consider the points at the entrance plane of DOE close to the optical axis, i.e. the term $\frac{\overrightarrow{r}^{\prime} \overrightarrow{u}^{\prime} }{c}$ in Eq. (12) can be neglected. The wavefront in the far-field close to the optical axis can be approximated by a plane wave, what allows us to use scalar fields in Eq. (12).

Next, to correctly describe the propagation of the field Eq. (12) through the DOE we assume that the Fresnel number *N*_*F*_ of DOE is large:

$${N}_{F}=\frac{{L}_{\perp }^{2}}{{\lambda }_{0}h}\gg 1,$$

where *λ*_0_ is the characteristic wavelength of the low-frequency oscillations, *h* is the thickness and *L*_⊥_ is the characteristic transverse size (e.g., radius) of DOE. This condition ensures that the diffraction in DOE is yet negligible, and the action of the DOE can be described by the multiplication of the field in $({\overrightarrow{r}}_{\perp },\omega )$ space by a transmission function:

13 $$T(\omega ,{\overrightarrow{r}}_{\perp })={e}^{-i\omega \tau ({\overrightarrow{r}}_{\perp })},$$

which represents the standard transfer function of the Fresnel diffraction for large Fresnel numbers *N*_*F*_^62^. Here the delay function $\tau ({\overrightarrow{r}}_{\perp })$ is related to the DOE thickness $h({\overrightarrow{r}}_{\perp })$ as:

$$\tau ({\overrightarrow{r}}_{\perp })=h({\overrightarrow{r}}_{\perp })\frac{n(\omega )-1}{c},$$

where *n*(*ω*) is the refractive index of the DOE. The transmission function Eq. (13) connects the field Eq. (12) just before the DOE with the field just behind the DOE. In this approximation, the DOE introduces a spatially-varying delay to the wavefront.

The dispersion inside the DOE can significantly influence the THz waveform. Nevertheless, the material of DOE is not obliged to have resonances near Ω_0_ (in contrast to the nonlinear layer), and thus a material with a flat dispersion can be chosen. Therefore, in the following consideration the dispersion in DOE is not taken into account. Nevertheless, since our THz pulses are broadband it is reasonable to set limits on the dispersion up to which the modifications in the waveshape of the THz pulse are still negligible. For this we must assume that the DOE width *h* ≪ *L*_*D*_, where ${L}_{D}={\tau }_{{\rm{\Sigma }}}^{2}/|{\beta }_{2}|$ is the dispersion length of the material for our typical pulse, *τ*_Σ_ is the duration of the generated THz waveforms, *β*_2_ = ∂^2^*k*/∂*ω*^2^ is the group-velocity dispersion, *k* is the wavenumber. Thus, the dispersion can be neglected if the group-velocity dispersion *β*_2_ in the vicinity Ω_0_ is: $|{\beta }_{2}|\ll {\tau }_{{\rm{\Sigma }}}^{2}/h$.

### The field at the observation point

The shape of the resulting waveform at the distant observation point is determined by the superposition of half-cycle pulses passing through the different parts of the DOE. In the Fraunhofer approximation such superposition is given by the following integral (up to a phase factor which we skip for clarity):

14 $$\begin{array}{lll}E(\overrightarrow{r},t) &  \sim  & {\int }_{\omega }{e}^{-i\omega (t-\frac{|\overrightarrow{r}|}{c})}d\omega {\iint }_{{\overrightarrow{r}}_{\perp }}E({\overrightarrow{r}}_{\perp },z={z}_{0}+h,\omega ){e}^{-i\frac{\omega }{c}\overrightarrow{u}{\overrightarrow{r}}_{\perp }}{d}^{2}{\overrightarrow{r}}_{\perp }\\  & = & {\iint }_{{\overrightarrow{r}}_{\perp }}{d}^{2}{\overrightarrow{r}}_{\perp }{\int }_{\omega }E({\overrightarrow{r}}_{\perp },z={z}_{0}+h,\omega ){e}^{-i\omega (t-\frac{|\overrightarrow{r}|}{c}+\frac{\overrightarrow{u}{\overrightarrow{r}}_{\perp }}{c})}d\omega n\\  & = & {\iint }_{{\overrightarrow{r}}_{\perp }}E({\overrightarrow{r}}_{\perp },z={z}_{0}+h,t-\frac{|\overrightarrow{r}|}{c}+\frac{\overrightarrow{u}{\overrightarrow{r}}_{\perp }}{c}){d}^{2}{\overrightarrow{r}}_{\perp },\end{array}$$

where $\overrightarrow{r}$ is the vector pointing to the observation point, $\overrightarrow{u}=\overrightarrow{r}/|\overrightarrow{r}|$ is the unit vector pointing in the same direction and the vector ${\overrightarrow{r}}_{\perp }$ lies in the initial plane, which is now located just behind the DOE.

Summarizing the equations (6), (12), (13) we  find for the field just behind the DOE (without regard to the lateral tails):

$$\overrightarrow{E}({\overrightarrow{r}}_{\perp },z={z}_{0}+h,t)=\tilde{E}{N}_{0}\sum _{k=0}^{1}\,{\sin {\rm{\Omega }}}_{0}(t-\frac{\tilde{D}}{c}-\tau ({\overrightarrow{r}}_{\perp })-k{\tau }_{p})\cdot {\rm{\Theta }}[t-\frac{\tilde{D}}{c}-\tau ({\overrightarrow{r}}_{\perp })-k{\tau }_{p}],$$

where *N*_0_ is the total number of oscillators in the layer. The field at the observation point Eq. (14) attains the form:

15 $$E(\overrightarrow{r},t) \sim \mathop{\iint }\limits_{{\overrightarrow{r}}_{\perp }}\,\tilde{E}{N}_{0}\sum _{k=0}^{1}\,{\sin {\rm{\Omega }}}_{0}(t-\tau ({\overrightarrow{r}}_{\perp })-\frac{\tilde{D}}{c}-\frac{|\overrightarrow{r}|}{c}+\frac{\overrightarrow{u}{\overrightarrow{r}}_{\perp }}{c}-k{\tau }_{p})\cdot {\rm{\Theta }}[t-\tau ({\overrightarrow{r}}_{\perp })-\frac{\tilde{D}}{c}-\frac{|\overrightarrow{r}|}{c}+\frac{\overrightarrow{u}{\overrightarrow{r}}_{\perp }}{c}-k{\tau }_{p}]{d}^{2}{\overrightarrow{r}}_{\perp }.$$

Eq. (15) implies that one can control the waveshape of the field at the detector by controlling of $\tau (\overrightarrow{r})$, that is, by imposing certain delay in the arrival of the partial half-sine waves.

## Shaping THz Waveforms with DOEs

### Optical wedge

We now consider particular DOEs which allow to shape the resulting THz radiation. We start by considering the simplest 1-D case (1D DOE) In this case we take the DOE in the form of a transparent wedge (see Fig. 1), the thickness of which varies linearly in the transverse direction (along *l*-axis with *l* = 0 corresponding to the wedge apex) according to the expression:

$$h(l)=l\cdot \,\tan \,\alpha ,$$

where *α* is the apex angle of the wedge. We assume that the wedge is transparent in the THz range and neglect the dispersion as discussed before. Assuming that the THz field is detected in far-field at the observation angle *ϕ* (see Fig. 1), we obtain the following expression for the total time delay in dependence on the transverse *l*-coordinate:

$$\tau (l)=\frac{h(l)}{c}\cdot (n+\frac{\sin (\varphi -\alpha )}{\sin \,\alpha })=\frac{l}{c}\cdot (n\,\tan \,\alpha +\frac{\sin (\varphi -\alpha )}{\cos \,\alpha }),$$

where *n* is the refractive index of the wedge at Ω_0_.

Based on Eq. (15), the electric field measured at the detector without regard to the weak lateral tails can be calculated as:

16 $$E(t)={E}_{0}{\int }_{0}^{L}\sum _{k=0}^{1}\sin [{{\rm{\Omega }}}_{0}{t}_{k}(l)]\cdot {\rm{\Theta }}({t}_{k}(l)){\rho }_{L}dl$$

with a scaling factor *E*_0_ and

$${t}_{k}(l)=t-\eta l-\frac{D}{c}-k{\tau }_{p},$$

where *L* is the length of the layer, *D* is the distance from the wedge to the observer, *ρ*_*L*_ is the linear density of medium oscillators and the parameter *η *is given as:

$$\eta =\frac{1}{c}\cdot (n\,\tan \,\alpha +\frac{\sin (\varphi -\alpha )}{\cos \,\alpha }).$$

The ananlytical solution of Eq. (16) is given in the Appendix I. Particularly, it is worth considering the value of the observation angle *ϕ*, given by the simple law of refraction. As can be easily seen, parameter *η* then turns to zero. From Appendix I one can see that for such case the field at the observation point represents just a half-sine wave times the total number of oscillators in the layer *N*_0_, since all partial waves after DOE propagate in-phase in this direction. However, for other values of the observation angle *ϕ* in the case when:

$$\eta L > {\tau }_{p},$$

the field at the observation point attains the form of a rectangular quasi-unipolar pulse with the amplitude:

17 $${E}_{m}=\frac{2{E}_{0}{\rho }_{L}}{\eta {{\rm{\Omega }}}_{0}}.$$

**Figure 6:**
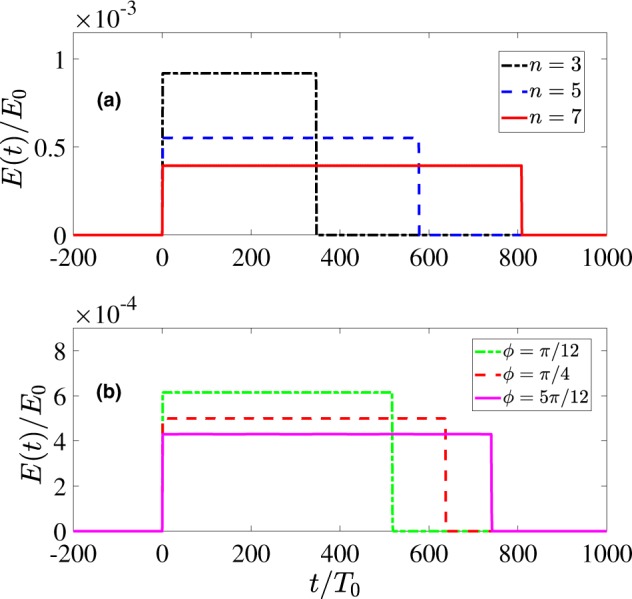
The response signal at a remote detector for a 1D wedge shown in Fig. 1. In (**a**) the curves for various values of the refractive index of the wedge *n* and *ϕ* = *π*/6 and in (**b**) for different values of *ϕ* for *n* = 5 are shown; The other parameters are: the layer width *h*_*L*_ = 6 mm, the eigen-period of the oscillators *T*_0_ = 10^−13^ s. *E*(*t*) is rescaled to the total number of oscillators in the layer *N*_0_ = *ρ*_*L*_*h*_*L*_. Insignificant low-amplitude long tails are neglected.

According to Eq. (17) *E*_*m*_ is dependent on *ϕ* and the refractive index *n*. The shapes of the resulting pulses in this case are illustrated in Fig. 6. In Fig. 6a we consider three different exemplary values of *n*. The apex angle of the wedge was chosen as *α* = *π*/6 and the angle *ϕ* was assumed to be *ϕ* = *π*/6. As the refractive index increases, the pulse duration also increases, whereas the amplitude decreases accordingly. In Fig. 6b we study the dependence of the pulse width on the observation angle *ϕ* for *n* = 5.0. Similar to Fig. 6a the duration of the pulses increases with the angle *ϕ* of the observer.

### Two-dimensional DOE

**Figure 7:**
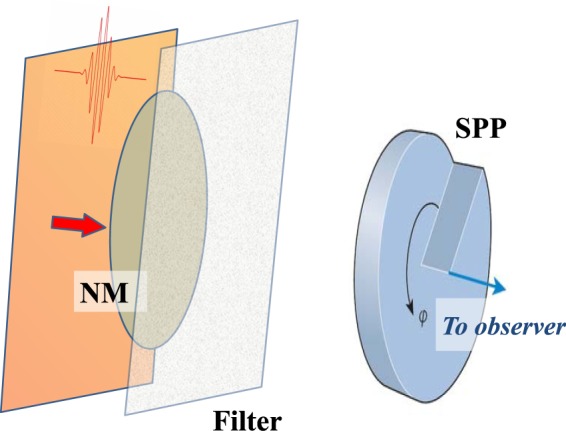
The scheme with 2D DOE. The nonlinear layer (NM) is excited by the pulses as before (for the clarity only one driving pulse is shown). The radiation emitted by NM oscillators is passed through a low-pass filter and then through a 2D DOE element — in this case a spiral-shaped phase plate (SPP).

Here we extend our method to more complex two-dimensional geometries shown in Fig. 7. We again excite the system with two pulses as shown in Fig. 2 separated by the delay *τ*_*p*_ = *T*_0_/2 and the resulting emission is passed through a DOE.

Proceeding analogously to the previous section we determine the field at the observation point as:

18 $$E(t)={E}_{0}\sum _{k=0}^{1}\,{\int }_{0}^{R}\,{\int }_{0}^{2\pi }\,\sin \,[{{\rm{\Omega }}}_{0}{t}_{k}(r,\phi )]{\rm{\Theta }}[{t}_{k}(r,\phi )]{\rho }_{S}rd\phi dr,$$

where *E*_0_ is a scaling factor, *ρ*_*S*_ is the surface density of medium oscillators, ${t}_{k}(r,\phi )=t-\frac{D}{c}-\tau (r,\phi )-k{\tau }_{p}$, *R* is the disk radius, *D* - distance from the DOE to the observation point (we may assume that the field is measured in the focal plane of a lens placed parallel to DOE) and *τ*(*r*, *φ*) is the delay due to DOE. If we take the observation point at the symmetry axis of the DOE, *τ*(*r*, *φ*) is given, to an inessential constant value, by:

19 $$\tau (r,\phi )=h(r,\phi )\frac{n-1}{c},$$

where the factor *h*(*r*, *φ*) describes the relief depth of the DOE and *n* is the refractive index of DOE at Ω_0_.

Assuming first the spiral phase plate (SPP) having the relief of the form:

$$h(r,\phi )={h}_{c}+\alpha \phi ,$$

that is, phase- but not radial-dependent, we obtain the following time delay function *τ*(*r*, *φ*) corresponding to the phase plate:

20 $$\tau (r,\phi )={\tau }_{c}+A\phi $$

with the constant *A*:

21 $$A=\alpha \frac{n-1}{c}.$$

Taking for definiteness the following parameter values: *n* = 3, *α* = 10 *μ*m/rad we can estimate the value of parameter *A* as *A* ~ 0.1 ps/rad.

**Figure 8:**
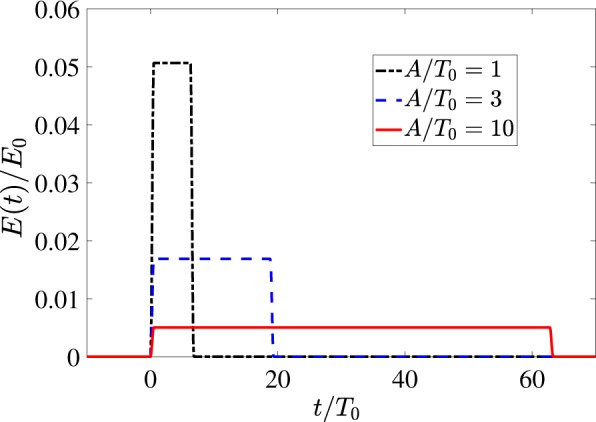
Rectangular pulses obtained for different values of the parameter *A* in Eq. (20) for *T*_0_ = 10^−13^ *s*. For convenience *E*(*t*) is rescaled for the total number of oscillators in the layer *N*_0_ = *πρ*_*S*_*R*^2^. Insignificant low-amplitude long tails are neglected.

Figure 8 shows the results of a numerical calculation of the field Eq. (18) for various values of the parameter *A* that corresponds to different relief depths of the phase plate. As one can see, the variation of the relief depth accessible with the DOE allows to produce rectangular quasi-unipolar pulses with a tunable duration ranging from hundreds femtoseconds to tens picoseconds.

**Figure 9:**
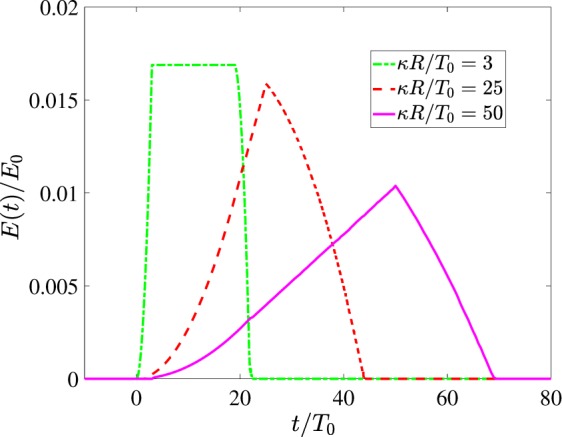
Subcycle pulses of various waveshape obtained for different values of *κ* from Eq. (22) for *T*_0_ = 10^−13^ *s*, *A*/*T*_0_ = 3, *R* = 1 cm. For convenience *E*(*t*) is rescaled for the total number of oscillators in the layer *N*_0_ = *πρ*_*S*_*R*^2^. Insignificant low-amplitude long tails are neglected.

By varying the delay function we can gain even more control over the waveshapes of the THz response. For instance, Fig. 9 shows the results of calculation of Eq. (18) for a more complex case of a helical axicon with

22 $$\tau (r,\phi )={\tau }_{c}+A\phi +\kappa r.$$

 In particular, depending on relative values of parameters *A* and *κ* we can obtain the continuous transition from the rectangular to triangular quasi-unipolar pulses (see Fig. 9 as well as Appendix II for more details).

## Conclusion

To summarize, we have proposed a new method for generation of subcycle THz pulses with unusual waveshapes such as rectangular or triangular ones. Our method is based on generation of half-oscillations of THz radiation in a nonlinear medium with a slow response with a pair of few-cycle pump pulses, and tuning the phase delay between different spatial parts of the resulting response using linear diffractive optics. According to our estimations, for high enough input powers in the tens of mJ range the amplitude of the generated field up to ≈100 kV/cm can be obtained. Although the threshold for THz generation is relatively low — in our simulations we needed intensities of 1 TW/cm^2^ or less, high pulse energies are required to achieve spatially broad pulses. The beam diameter must be in every case much larger that the THz wavelength and thus must be at least in mm range. More generally, one can ask if we can produce any required waveshape by tuning the phase delay *h*(*r*, *φ*) of DOE. This more general question is beyond the scope of this paper but there are no obvious reasons why it could not be possible. Moreover, by tuning the DOE one might be able to produce tunable THz pulses from more convenient and less broadband THz responses than considered here.

Thus, the results of the present work extend the possibility to generate customary waveshapes to the terahertz range. Such customary shapes of the electric field (rather than the slow envelope) was up to now a prerogative of electronics. We believe that such new possibility will be useful in ultrafast optical information processing. Furthermore, the method can be upscaled to the optical range if the strong attosecond pump pulses are used.

## Supplementary information

File with supplementary materials

## References

[1] Roskos HG, Thomson MD, Kress M, Löffler T (2007). Broadband THz emission from gas plasmas induced by femtosecond optical pulses: From fundamentals to applications. Laser Photon. Rev..

[2] Reimann K (2007). Table-top sources of ultrashort THz pulses. Rep. Progr. Phys..

[3] Bartel T, Gaal P, Reimann K, Woerner M, Elsaesser T (2005). Generation of single-cycle THz transients with high electric-field amplitudes. Opt. Lett..

[4] Babushkin I, Skupin S, Herrmann J (2010). Generation of terahertz radiation from ionizing two-color laser pulses in Ar filled metallic hollow waveguides. Opt. Expr..

[5] Lepeshov S, Gorodetsky A, Krasnok A, Rafailov E, Belov P (2017). Enhancement of terahertz photoconductive antenna operation by optical nanoantennas. Laser Photon. Rev..

[6] Jepsen PU, Cooke DG, Koch M (2011). Terahertz spectroscopy and imaging–Modern techniques and applications. Laser Photon. Rev..

[7] Parrott EP, Zeitler JA (2015). Terahertz time-domain and low-frequency Raman spectroscopy of organic materials. Applied spectroscopy.

[8] Fülöp JA (2014). Efficient generation of THz pulses with 0.4 mJ energy. Opt. Expr..

[9] Nugraha PS (2019). Demonstration of a tilted-pulse-front pumped plane-parallel slab terahertz source. Opt. Lett..

[10] Fülöp JA (2016). Highly efficient scalable monolithic semiconductor terahertz pulse source. Optica.

[11] Blanchard F (2014). Terahertz pulse generation from bulk GaAs by a tilted-pulse-front excitation at 1.8 *μ*m. Appl. Phys. Lett..

[12] Vicario C (2014). Generation of 0.9-mJ THz pulses in DSTMS pumped by a Cr:Mg_2_SiO_4_ laser. Opt. Lett..

[13] Lu J (2018). Efficient terahertz generation in highly nonlinear organic crystal HMB-TMS. Opt. Expr..

[14] Babushkin I (2017). Terahertz and higher-order Brunel harmonics: from tunnel to multiphoton ionization regime in tailored fields. J. Mod. Opt..

[15] Kim KY, Glownia JH, Taylor AJ, Rodriguez G (2007). Terahertz emission from ultrafast ionizing air in symmetry-broken laser fields. Opt. Expr..

[16] Kim KY, Taylor AJ, Glownia JH, Rodriguez G (2008). Coherent control of terahertz supercontinuum generation in ultrafast laser-gas interactions. Nature Photon..

[17] Babushkin I (2011). Tailoring terahertz radiation by controlling tunnel photoionization events in gases. New J. Phys..

[18] Berge L, Skupin S, Köhler C, Babushkin I, Herrmann J (2013). 3D Numerical Simulations of THz Generation by Two-Color Laser Filaments. Phys. Rev. Lett..

[19] Lepeshov S (2018). Boosting Terahertz Photoconductive Antenna Performance with Optimised Plasmonic Nanostructures. Sci. Rep..

[20] Krausz F, Ivanov M (2009). Attosecond physics. Rev. Mod. Phys..

[21] Wu H-C, Meyer-ter-Vehn J (2012). Giant half-cycle attosecond pulses. Nature Photon..

[22] Xu J (2018). Terawatt-scale optical half-cycle attosecond pulses. Sci. Rep..

[23] Hassan MT (2016). Optical attosecond pulses and tracking the nonlinear response of bound electrons. Nature.

[24] Arkhipov MV (2017). Generation of unipolar half-cycle pulses via unusual reflection of a single-cycle pulse from an optically thin metallic or dielectric layer. Opt. Lett..

[25] Ramasesha K, Leone SR, Neumark DM (2016). Real-time probing of electron dynamics using attosecond time-resolved spectroscopy. Annu. Rev. Phys. Chem..

[26] Corkum PB, Krausz F (2007). Attosecond science. Nature physics.

[27] Calegari F (2016). Advances in attosecond science. J. Phys. B.

[28] Arkhipov RM (2019). Unipolar subcycle pulse-driven nonresonant excitation of quantum systems. Opt. Lett..

[29] Arkhipov RM (2017). Generation of unipolar pulses in nonlinear media. JETP Lett..

[30] Bullough RK, Ahmad F (1971). Exact Solutions of the Self-Induced Transparency Equations. Phys. Rev. Lett..

[31] Kalosha VP, Herrmann J (1999). Formation of optical subcycle pulses and full Maxwell-Bloch solitary waves by coherent propagation effects. Phys. Rev. Lett..

[32] Kaplan AE, Shkolnikov PL (1995). Electromagnetic â€œbubblesâ€ and shock waves: unipolar, nonoscillating EM solitons. Phys. Rev. Lett..

[33] Song X, Yang W, Zeng Z, Li R, Xu Z (2010). Unipolar half-cycle pulse generation in asymmetrical media with a periodic subwavelength structure. Phys. Rev. A.

[34] Vysotina NV, Rosanov NN, Semenov VE (2009). Extremely short dissipative solitons in an active nonlinear medium with quantum dots. Opt. Spectr..

[35] Leblond H (2008). Half-cycle optical soliton in quadratic nonlinear media. Phys. Rev. A.

[36] Kozlov VV, Rosanov NN, Angelis CD, Wabnitz S (2011). Generation of unipolar pulses from nonunipolar optical pulses in a nonlinear medium. Phys. Rev. A.

[37] Leblond H, Mihalache D (2013). Models of few optical cycle solitons beyond the slowly varying envelope approximation. Phys. Rep..

[38] Arkhipov RM, Arkhipov MV, Babushkin I, Belov PA, Tolmachev YA (2016). Generation of unipolar optical pulses in a Raman-active medium. Laser Phys. Lett..

[39] Arkhipov AV (2016). Few-cycle pulse-driven excitation response of resonant medium with nonlinear field coupling. Laser Phys. Lett..

[40] Pakhomov AV (2017). All-optical control of unipolar pulse generation in a resonant medium with nonlinear field coupling. Phys. Rev. A.

[41] Bessonov EG (1981). On a class of electromagnetic waves. Sov. Phys. JETP.

[42] Bessonov EG (1991). Conventionally strange electromagnetic waves. Nucl. Instr. and Meth. A.

[43] Weiner AM (2011). Ultrafast optical pulse shaping: A tutorial review. Opt. Commun..

[44] Mendis R, Nag A, Chen F, Mittleman DM (2010). A tunable universal terahertz filter using artificial dielectrics based on parallel-plate waveguides. Appl. Phys. Lett..

[45] Kawada Y, Yasuda T, Takahashi H (2016). Carrier envelope phase shifter for broadband terahertz pulses. Opt. Lett..

[46] Gingras L, Cooke DG (2017). Direct temporal shaping of terahertz light pulses. Optica.

[47] Gingras L, Cui W, Schiff-Kearn AW, Ménard JM (2018). Active phase control of terahertz pulses using a dynamic waveguide. Opt. Expr..

[48] Nugraha PS (2018). Efficient semiconductor multicycle terahertz pulse source. J. Phys. B.

[49] Lu J (2015). Tunable multi-cycle THz generation in organic crystal HMQ-TMS. Opt. Expr..

[50] Allen, L., Eberly, J. H. *Optical Resonance and Two-Level Atoms* (John Wiley & Sons Inc., New York, 1975).

[51] Arkhipov RM (2018). Generation of Extremely Short Pulses upon Excitation of a Resonant Medium by a Superluminal Light Spot. Opt. Spectr..

[52] Ziguleva DO (2018). Laser beam deflector based generation of few-cycle electromagnetic pulses in a circular nonlinear medium. Opt. Commun..

[53] Platonenko VT, Khokhlov RV (1964). On the Mechanism of Operation of a Raman Laser. Sov. Phys. JETP.

[54] Akhmanov, S. A., Nikitin, S. Yu. *Physical Optics* (Nauka, Moscow, 2004; Clarendon, Oxford, 1997).

[55] Ginzburg P (2012). Nonlinearly coupled localized plasmon resonances: Resonant second-harmonic generation. Phys. Rev. B.

[56] Novotny L, van Hulst N (2011). Antennas for light. Nature Photon..

[57] Biagioni P, Huang J-S, Hecht B (2012). Nanoantennas for visible and infrared radiation. Rep. Prog. Phys..

[58] Bera D, Qian L, Tseng TK, Holloway PH (2010). Quantum dots and their multimodal applications: a review. Materials.

[59] Yao X, Tokman M, Belyanin A (2014). Efficient Nonlinear Generation of THz Plasmons in Graphene and Topological Insulators. Phys. Rev. Lett..

[60] Hartmann RR, Kono J, Portnoi ME (2014). Terahertz science and technology of carbon nanomaterials. Nanotechn..

[61] Chen S (2014). Broadband optical and microwave nonlinear response in topological insulator. Opt. Mater. Expr..

[62] Born, M., Wolf, E. *Principles of optics* (Pergamon Press, 1980).

