# Supplementary material for: Unusual terahertz waveforms from a resonant medium controlled by diffractive optical elements
Source: Sci Rep. 2019 May 15;9:7444. doi: 10.1038/s41598-019-43852-w (PMC6520407; doi:10.1038/s41598-019-43852-w)
Supplement: Supplementary file 1 — File with supplementary materials [file 41598_2019_43852_MOESM1_ESM.pdf]

Unusual terahertz waveforms from a resonant medium controlled by diffractive optical elements

A.V. Pakhomov[1,2,*], R.M. Arkhipov[1,2], M.V. Arkhipov[2], A. Demircan[3,4], U. Morgner[3,4], N.N. Rosanov[2,5,6], and I. Babushkin[3,4,7]

[1]St. Petersburg State University, 7/9 Universitetskaya nab., St. Petersburg, 199034 Russia
[2]ITMO University, Kronverkskiy Prospekt 49, St. Petersburg 197101, Russia
[3]Institute of Quantum Optics, Welfengarten 1, 30167 Hannover, Germany
[4]Cluster of Excellence PhoenixD (Photonics, Optics, and Engineering – Innovation Across Disciplines), Hannover, Germany
[5]Ioffe Institute, Politekhnicheskaya str. 26, St. Petersburg 194021, Russia
[6]Vavilov State Optical Institute, Kadetskaya Liniya v.o. 5/2, St. Petersburg 199053, Russia
[7]Max Born Institute, Max-Born-Strasse 2a, Berlin 10117, Germany
[*]Corresponding author: antpakhom@gmail.com

Appendix I. Analytical solution of Eq. (16)

We provide here an exact solution of the integral (16). Two cases should be distinguished here depending on the relative values of ηL (see Eq. (17)) and $\tau_p = T_0/2$. We start with the assumption that $\eta L > \tau_p$. If we denote $t'' = t - \frac{D}{c}$, at the time $0 < t'' < \tau_p$ only first pulse is acting on the medium, and the electric field of the response can be obtained as

$$E(t) = E_0 \int_0^{t''/\eta} \sin[\Omega_0(t'' - \eta l)]\rho_L dl = \frac{E_0 \rho_L}{\eta \Omega_0}\Big(1 - \cos[\Omega_0 t'']\Big).$$

The amplitude of the electric field increases from zero at $t'' = 0$ to the maximum value

$$E_m = \frac{2E_0\rho_L}{\eta\Omega_0}$$

at $t'' = \tau_p$.

At the time $\tau_p < t'' < \eta L$ the detected electric field results from the interference of the half-waves emitted by the layer and is expressed as

$$\begin{aligned} E(t) &= E_0\Big(\int_0^{t''/\eta} \sin[\Omega_0(t''-\eta l)]\rho_L dl + \int_0^{(t''-\tau_p)/\eta} \sin[\Omega_0(t''-\eta l - \tau_p)]\rho_L dl\Big) \\ &= \frac{E_0\rho_L}{\eta\Omega_0}\Big(2 - \cos(\Omega_0(t''-\tau_p)) - \cos(\Omega_0 t'')\Big). \end{aligned}$$

Since $\tau_p = \frac{T_0}{2}$, the electric field attains the constant value equal to $\frac{2E_0\rho_L}{\eta\Omega_0}$.

For $\eta L < t'' < \eta L + \tau_p$ one detects only the sum of the trailing edges of the emitted half-waves and the electric field of the response is now given as

$$\begin{aligned} E(t) &= E_0\Big(\int_0^{L} \sin[\Omega_0(t''-\eta l)]\rho_L dl + \int_0^{(t''-\tau_p)/\eta} \sin[\Omega_0(t''-\eta l - \tau_p)]\rho_L dl\Big) \\ &= \frac{E_0\rho_L}{\eta\Omega_0}\Big(\cos[\Omega_0(t''-\eta L)] - \cos[\Omega_0 t''] + 1 - \cos[\Omega_0(t''-\tau_p)]\Big) \\ &= \frac{E_0\rho_L}{\eta\Omega_0}\Big(1 + \cos[\Omega_0(t''-\eta L)]\Big). \end{aligned}$$

The amplitude decreases from $\frac{2E_0\rho_L}{\eta\Omega_0}$ at $t'' = \eta L$ to zero at $t'' = \eta L + \tau_p$.

If $t'' > \eta L + \tau_p$ the electric field can be defined as

$$E(t) = E_0\Big(\int_0^L \sin(\Omega_0(t'' - \eta l))\rho_L dl + \int_0^L \sin(\Omega_0(t - \eta l - \tau_p))\rho_L dl\Big)$$
$$= \frac{E_0\rho_L}{\eta\Omega_0}\Big(\cos(\Omega_0(t'' - \eta L)) - \cos(\Omega_0 t'') + \cos(\Omega_0(t'' - \eta L - \tau_p)) - \cos(\Omega_0(t'' - \tau_p))\Big).$$

Since $\tau_p = \frac{T_0}{2}$ this expression becomes zero.
The total pulse duration is thus

$$\tau_{total} = \eta L + \tau_p.$$

Let us assume now that $\eta L < \tau_p$. Then for $0 < t'' < \eta L$ only first pulse moves along the layer and the electric field grows as

$$E(t) = E_0 \int_0^{t''/\eta} \sin[\Omega_0(t'' - \eta l)]\rho_L dl = \frac{E_0\rho_L}{\eta\Omega_0}\Big(1 - \cos[\Omega_0 t'']\Big).$$

When $\eta L < t'' < \tau_p$ we get:

$$E(t) = E_0 \int_0^L \sin[\Omega_0(t'' - \eta l)]\rho_L dl = \frac{E_0\rho_L}{\eta\Omega_0}\Big(\cos[\Omega_0(t'' - \eta L)] - \cos[\Omega_0 t'']\Big)$$
$$= \frac{2E_0\rho_L}{\eta\Omega_0}\sin[\Omega_0(t'' - \eta L/2)]\sin[\Omega_0\eta L/2],$$

i.e. instead of flat-top pulse we obtain sinusoidal pulse shape also in its central part.
For $\tau_p < t'' < \eta L + \tau_p$ the field appears to be:

$$E(t) = E_0\Big(\int_0^L \sin[\Omega_0(t'' - \eta l)]\rho_L dl + \int_0^{(t'' - \tau_p)/\eta} \sin[\Omega_0(t'' - \eta l - \tau_p)]\rho_L dl\Big)$$
$$= \frac{E_0\rho_L}{\eta\Omega_0}\Big(\cos[\Omega_0(t'' - \eta L)] - \cos[\Omega_0 t''] + 1 - \cos[\Omega_0(t'' - \tau_p)]\Big) = \frac{E_0\rho_L}{\eta\Omega_0}\Big(1 + \cos[\Omega_0(t'' - \eta L)]\Big).$$

Finally, for $t'' > \eta L + \tau_p$ the electric field turns to zero similar to the previous case.

Appendix II. Analytical solution of Eq. (18) for the axicon Eq. (22)

Let us now compute the electric field for the helical axicon, given by Eq. (22). To simplify the calculation we will make the following assumption:

$$2\pi A, \kappa R \gg \frac{T_0}{2}. \tag{1}$$

This means that the additional delay introduced by DOE is much larger than the duration of the quasi-unipolar pulses obtained directly from the nonlinear medium. The assumption Eq. (1) looks quite natural since DOE is intended to significantly stretch the incident pulses.

With this assumption, the integral Eq. (18) can be calculated in simple analytical form. Indeed, we can now neglect the contribution from the trailing and leading edges of the rectangular pulses resulting from the integration over polar angle φ in Eq. (18). Then these rectangular pulses are assumed to have sharp edges.

We will consider in the following two different cases. In the first case, we take $2\pi A > \kappa R + \frac{T_0}{2}$. Then with the aforesaid assumption we obtain that for $\tau_c < t'' < \tau_c + \frac{T_0}{2} + \kappa R$ the electric field can be defined as:

$$E(t) = E_0 \int_0^{\frac{t'' - \frac{T_0}{2}}{\kappa}} \frac{2}{\Omega_0 A}\rho_S r dr = E_0\rho_S \frac{(t'' - \frac{T_0}{2})^2}{\kappa^2\Omega_0 A},$$

where we denoted again $t'' = t - \frac{D}{c}$. One can see that the field amplitude increases from 0 to $\frac{E_0\rho_S R^2}{\Omega_0 A}$.

If $\tau_c + \frac{T_0}{2} + \kappa R < t'' < \tau_c + 2\pi A$ the electric field is constant and given as:

$$E(t) = E_0 \int_0^R \frac{2}{\Omega_0 A}\rho_S r dr = E_0 \frac{\rho_S R^2}{\Omega_0 A}.$$

At last, for $\tau_c + 2\pi A < t'' < \tau_c + 2\pi A + \kappa R$ one obtains:

$$E(t) = E_0 \int_{\frac{t''-2\pi A}{\kappa}}^{R} \frac{2}{\Omega_0 A} \rho_S r dr = E_0 \rho_S \frac{\kappa^2 R^2 - (t'' - 2\pi A)^2}{\kappa^2 \Omega_0 A},$$

i.e. the electric field decreases from $E = \frac{E_0 \rho_S R^2}{\Omega_0 A}$ at $t'' = 2\pi A$ to $E = 0$ at $t'' = 2\pi A + \kappa R$.

When $t'' > \tau_c + 2\pi A + \kappa R$ the expression under the integral sign in Eq. (18) vanishes and hence the electric field equals zero.

In the second case, we take $\kappa R + \frac{T_0}{2} > 2\pi A$. Then if $\tau_c + \frac{T_0}{2} < t'' < \tau_c + 2\pi A$ the electric field is given as:

$$E(t) = E_0 \int_0^{\frac{t''-\frac{T_0}{2}}{\kappa}} \frac{2}{\Omega_0 A} \rho_S r dr = E_0 \rho_S \frac{(t'' - \frac{T_0}{2})^2}{\kappa^2 \Omega_0 A},$$

so the amplitude increases from $E = 0$ at $t'' = \tau_c + \frac{T_0}{2}$ to $E = E_0 \rho_S \frac{(2\pi A - \frac{T_0}{2})^2}{\kappa^2 \Omega_0 A}$ at $t'' = \tau_c + 2\pi A$.

For $\tau_c + 2\pi A < t'' < \tau_c + \frac{T_0}{2} + \kappa R$ the electric field is:

$$E(t) = E_0 \int_{\frac{t''-2\pi A}{\kappa}}^{\frac{t''-\frac{T_0}{2}}{\kappa}} \frac{2}{\Omega_0 A} \rho_S r dr = E_0 \rho_S \frac{(t'' - \frac{T_0}{2})^2 - (t'' - 2\pi A)^2}{\kappa^2 \Omega_0 A} =$$

$$E_0 \rho_S \frac{t''(4\pi A - T_0) + (\frac{T_0^2}{4} - 4\pi^2)}{\kappa^2 \Omega_0 A},$$

what means that electric field linearly grows from $E = E_0 \rho_S \frac{(2\pi A - \frac{T_0}{2})^2}{\kappa^2 \Omega_0 A}$ at $t'' = \tau_c + 2\pi A$ to $E = E_0 \rho_S \frac{\kappa^2 R^2 - (\kappa R - 2\pi A + \frac{T_0}{2})^2}{\kappa^2 \Omega_0 A}$ at $t'' = \tau_c + \frac{T_0}{2} + \kappa R$.

Finally, for $\tau_c + \frac{T_0}{2} + \kappa R < t'' < \tau_c + 2\pi A + \kappa R$:

$$E(t) = E_0 \int_{\frac{t''-2\pi A}{\kappa}}^{R} \frac{2}{\Omega_0 A} \rho_S r dr = E_0 \rho_S \frac{\kappa^2 R^2 - (t'' - 2\pi A)^2}{\kappa^2 \Omega_0 A},$$

so electric field amplitude decreases from $E = E_0 \rho_S \frac{\kappa^2 R^2 - (\kappa R - 2\pi A + \frac{T_0}{2})^2}{\kappa^2 \Omega_0 A}$ at $t'' = \tau_c + \frac{T_0}{2} + \kappa R$ to $E = 0$ at $t'' = \tau_c + 2\pi A + \kappa R$.

For $t'' > \tau_c + 2\pi A + \kappa R$ electric field identically equals zero.

Appendix III. Effect of the lateral tails on the waveform of the produced pulse

In order to find out the role of the lateral tails on the pulse shape, we should first notice that the durations of these tails are several orders of magnitude larger than the duration of the central half-sine wave. Thus we can approximately assume, that the amplitudes of the tails just slightly change over the time interval, equal to the duration of the total THz pulse.

Therefore, in a first approximation, we can describe the tails as having some constant amplitude $E_0 \Delta a$ with $\Delta a \ll 1$, which is negative in sign. Then, for example, Eq. (18) attains the form:

$$E(t) = E_0 \int_0^R \int_0^{2\pi} \Big(\sum_{k=0}^{1} \sin[\Omega_0 t_k(r,\varphi)] \Theta[t_k(r,\varphi)] - \Delta a \Big) \rho_S r d\varphi dr = E^0(t) - \Delta E,$$

where $E^0(t)$ is the field obtained in Appendix II without taking tails into account and the additional term ΔE attributed to the contribution from lateral tails appears to be a constant $\Delta E = \pi \Delta a \rho_S R^2$. One can hence conclude that the lateral tails lead just to the downward shift of the produced pulse for the constant value ΔE without changing the pulse shape.

In a similar way we can find for Eq. (16):

$$E(t) = E_0 \int_0^L \Big(\sum_{k=0}^{1} \sin[\Omega_0 t_k(l)] \cdot \Theta(t_k(l)) - \Delta a \Big) \rho_L dl = E^0(t) - \Delta E,$$

with constant downward shift of the field for $\Delta E = \Delta a \rho_L L$.
